# A roadmap for gene functional characterisation in crops with large genomes: Lessons from polyploid wheat

**DOI:** 10.7554/eLife.55646

**Published:** 2020-03-24

**Authors:** Nikolai M Adamski, Philippa Borrill, Jemima Brinton, Sophie A Harrington, Clémence Marchal, Alison R Bentley, William D Bovill, Luigi Cattivelli, James Cockram, Bruno Contreras-Moreira, Brett Ford, Sreya Ghosh, Wendy Harwood, Keywan Hassani-Pak, Sadiye Hayta, Lee T Hickey, Kostya Kanyuka, Julie King, Marco Maccaferrri, Guy Naamati, Curtis J Pozniak, Ricardo H Ramirez-Gonzalez, Carolina Sansaloni, Ben Trevaskis, Luzie U Wingen, Brande BH Wulff, Cristobal Uauy

**Affiliations:** 1 John Innes Centre, Norwich Research ParkNorwichUnited Kingdom; 2 School of Biosciences, University of BirminghamBirminghamUnited Kingdom; 3 John Bingham LaboratoryCambridgeUnited Kingdom; 4 Commonwealth Scientific and Industrial Research Organisation, Agriculture and Food (CSIRO)CanberraAustralia; 5 Council for Agricultural Research and Economics, Research Centre for Genomics and BioinformaticsFiorenzuola d'ArdaItaly; 6 European Molecular Biology Laboratory, European Bioinformatics Institute, Wellcome Genome CampusHinxtonUnited Kingdom; 7 Rothamsted ResearchHarpendenUnited Kingdom; 8 Queensland Alliance for Agriculture and Food Innovation, The University of QueenslandSt LuciaAustralia; 9 Division of Plant and Crop Sciences, The University of Nottingham, Sutton Bonington CampusLoughboroughUnited Kingdom; 10 Department of Agricultural and Food Sciences (DISTAL), Alma Mater Studiorum - Università di Bologna (University of Bologna)BolognaItaly; 11 Crop Development Centre, University of SaskatchewanSaskatoonCanada; 12 International Maize and Wheat Improvement Center (CIMMYT)El BatánMexico; University of LausanneSwitzerland; University of LausanneSwitzerland

**Keywords:** crop genetics, genomics, wheat, polyploidy

## Abstract

Understanding the function of genes within staple crops will accelerate crop improvement by allowing targeted breeding approaches. Despite their importance, a lack of genomic information and resources has hindered the functional characterisation of genes in major crops. The recent release of high-quality reference sequences for these crops underpins a suite of genetic and genomic resources that support basic research and breeding. For wheat, these include gene model annotations, expression atlases and gene networks that provide information about putative function. Sequenced mutant populations, improved transformation protocols and structured natural populations provide rapid methods to study gene function directly. We highlight a case study exemplifying how to integrate these resources. This review provides a helpful guide for plant scientists, especially those expanding into crop research, to capitalise on the discoveries made in *Arabidopsis* and other plants. This will accelerate the improvement of crops of vital importance for food and nutrition security.

## Introduction

Research in *Arabidopsis* and other model species has uncovered mechanisms regulating important biological processes in plants. However, as research in these model species does not always translate directly into crop species such as wheat, understanding gene function in crop species themselves is critical for crop improvement. With the advent of functional genomics resources in wheat and other crops, discoveries from model species can rapidly be tested and functional genetic studies can now be performed for agronomically-important traits directly in the crops themselves (Borrill, 2020).

**Figure 1.**
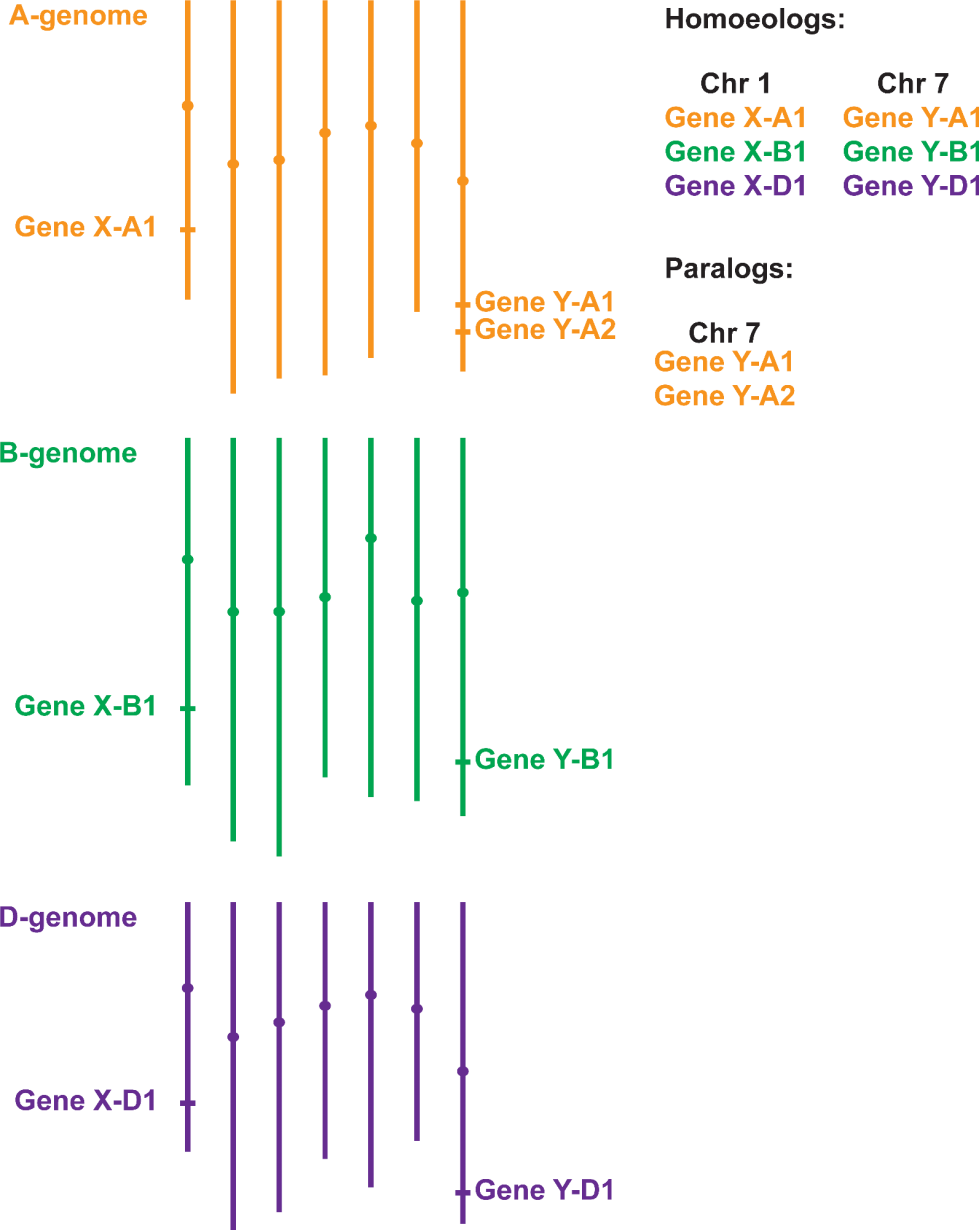
Gene homology within polyploid wheat. Due to two separate hybridisation events, genes in polyploid wheat will be present in multiple copies called homoeologs, which usually have similar chromosome locations. In the example of hexaploid bread wheat illustrated here, Gene X has homoeologs on chromosomes 1A, 1B and 1D. Duplicated genes, called paralogs (e.g. two copies of Gene Y on chromosome 7A), have evolved either within wheat or in one of its ancestral species. Most paralogs arise from intra-chromosomal duplications, although inter-chromosomal duplications can also occur.

The most common forms of domesticated wheat are tetraploid durum wheat (*Triticum turgidum* ssp. *durum* L.) and hexaploid bread wheat (*Triticum aestivum L.*). Polyploid wheat is derived from hybridisation events between different ancestral progenitor species reviewed in Matsuoka (2011), and thus each gene typically exists as two (tetraploid durum wheat) or three (hexaploid bread wheat) copies. These closely related copies, known as homoeologous genes, are on average >95% similar across their coding regions (Figure 1) and usually have a highly conserved gene structure. Tetraploid and hexaploid wheat have large genomes, 12 and 16 Gbp respectively, which consist mostly (>85%) of repetitive elements. The combination of these factors has, for a long time, hampered the development of genomics tools in wheat and other crops with large genomes, such as sugarcane (Garsmeur et al., 2018). Recent advances in sequencing technologies and bioinformatics tools has helped overcome these difficulties, and there are now a wide range of resources available for genomic analysis in wheat. The speed of wheat research has also been limited by its relatively long generation time, which ranges from four to six months depending on the requirement of cold periods (vernalisation) to induce flowering. Again, recent advances in the use of controlled growth conditions have radically changed these timeframes (Watson et al., 2018). Wheat has now become a tractable system for translational, comparative and functional genomics (Borrill et al., 2019).

**Figure 2.**
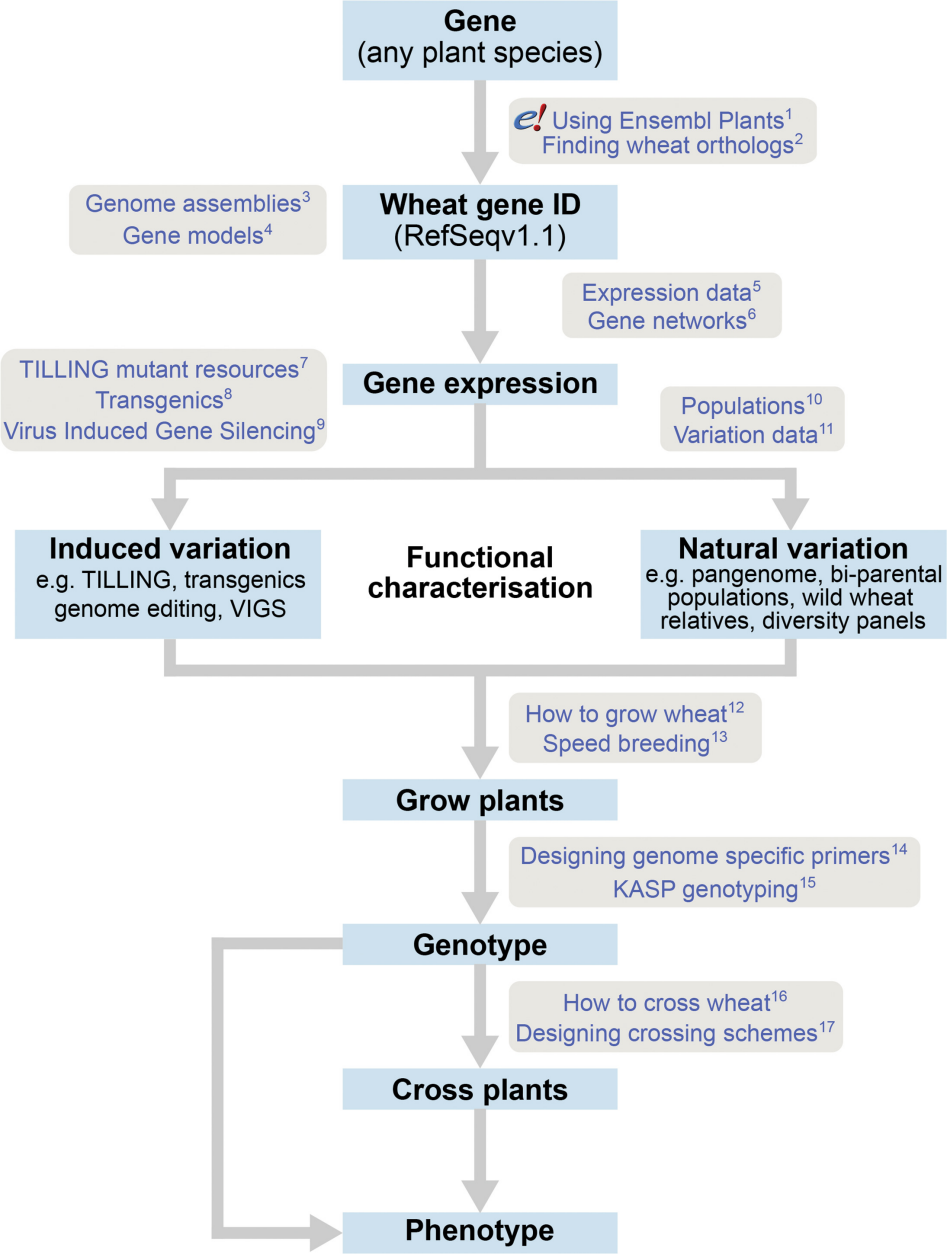
The roadmap for gene characterisation in wheat. Overview of a proposed strategy to take a gene from any plant species, identify the correct wheat ortholog(s) using Ensembl Plants (https://plants.ensembl.org) and determine gene expression using expression browsers and gene networks. Suggestions for functional characterisation are provided including induced variation such as mutants, transgenics or Virus-Induced Gene Silencing (VIGs). In addition, publicly available populations incorporating natural variation are available. Finally steps for growing, genotyping and crossing plants are outlined. Links to detailed tutorials and further information are provided and can be found on www.wheat-training.com. (1) www.wheat-training.com/wp-content/uploads/Genomic_resources/pdfs/EnsemblPlants-primer.pdf (2) www.wheat-training.com/wp-content/uploads/Genomic_resources/pdfs/Finding-wheat-orthologs.pdf (3) www.wheat-training.com/wp-content/uploads/Genomic_resources/pdfs/Genome_assemblies.pdf (4) www.wheat-training.com/wp-content/uploads/Genomic_resources/pdfs/Gene-models.pdf (5) www.wheat-training.com/wp-content/uploads/Genomic_resources/pdfs/Expression-browsers.pdf (6) www.wheat-training.com/wp-content/uploads/Genomic_resources/pdfs/Gene-networks.pdf (7) www.wheat-training.com/wp-content/uploads/Functional_studies/PDFs/Selecting-TILLING-mutants.pdf (8) www.wheat-training.com/wp-content/uploads/Functional_studies/PDFs/Transgenics.pdf (9) www.wheat-training.com/wp-content/uploads/Functional_studies/PDFs/Virus_Induced_Gene_Silencing.pdf (10) www.wheat-training.com/wp-content/uploads/Functional_studies/PDFs/Populations.pdf (11) www.wheat-training.com/wp-content/uploads/Genomic_resources/Variation-data.pdf (2) www.wheat-training.com/wp-content/uploads/Wheat_growth/pdfs/Growing_Wheat_final.pdf (13) www.wheat-training.com/wp-content/uploads/Wheat_growth/pdfs/Speed_breeding.pdf (14) www.wheat-training.com/wp-content/uploads/Functional_studies/PDFs/Designing-genome-specific-primers.pdf (15) https://www.biosearchtech.com/support/education/kasp-genotyping-reagents/running-kasp-genotyping-reactions (16) http://www.wheat-training.com/wp-content/uploads/Wheat_growth/pdfs/How-to-cross-wheat-pdf.pdf (17) www.wheat-training.com/wp-content/uploads/Functional_studies/PDFs/Designing-crossing-schemes.pdf.

Here we describe some of the recent developments in wheat genomics, focussing on published and publicly available resources and tools, and lay out a roadmap for their use (Figure 2). We present available wheat genome assemblies and annotations and discuss a series of approaches to functionally characterise genes. We also outline strategies for growing, crossing and genotyping wheat using the latest available tools and techniques. Finally, we present a case study that encapsulates the above steps and highlights potential pitfalls. We focus mainly on the Ensembl Plants database, as it integrates many of the publicly available data on wheat. However, other databases such as URGI (https://wheat-urgi.versailles.inra.fr/; Alaux et al., 2018), the Wheat Information System (WheatIS; http://www.wheatis.org/), and GrainGenes (https://wheat.pw.usda.gov/GG3/; Blake et al., 2019) also host and integrate similar, but complementary, genetic, genomic and phenomic data for wheat. We expect this review will be a helpful guide for plant scientists who already work on wheat or who are considering expanding their research into crops with large genomes such as wheat.

## Wheat genome assemblies

A high-quality genome reference sequence is an essential resource for functional genetics and genomics in any species. Several hexaploid wheat genome assemblies have been released over the past eight years (Brenchley et al., 2012; Mayer et al., 2014; Chapman et al., 2015; Clavijo et al., 2017; Zimin et al., 2017). The most comprehensive assembly, called RefSeqv1.0, is a chromosome-level genome assembly annotated with high and low confidence gene models (The International Wheat Genome Sequencing Consortium (IWGSC) et al., 2018). Two tetraploid wheat genomes have also been sequenced, assembled, and annotated to the same standard as RefSeqv1.0 — the wild tetraploid progenitor of wheat, wild emmer (Avni et al., 2017), and a modern durum wheat variety (Maccaferri et al., 2019). Diploid ancestral progenitor species have also been assembled to varying levels of completeness (Luo et al., 2017; Zhao et al., 2017; Ling et al., 2018; Miki et al., 2019). We summarise the annotated assemblies for polyploid wheat in Table 1; in this review we will focus mainly on the RefSeqv1.0 assembly.

**Table 1.** Comparison of annotated genome assemblies in hexaploid and tetraploid wheat.

	CSS	TGACv1	RefSeqv1.0	Durum wheat	Wild emmer wheat
Publication	Mayer et al., 2014	Clavijo et al., 2017	The International Wheat Genome Sequencing Consortium (IWGSC) et al., 2018	Maccaferri et al., 2019	Avni et al., 2017
Contigs/Chromosomes	>1 million	735,943	21 chromosomes + ChrU	14 chromosomes + ChrU	14 chromosomes + ChrU
Mean scaffold size	7.7 kbp	88.7 kbp	Chromosomes	Chromosomes	Chromosomes
Assembly Size	10.2 Gbp	13.4 Gbp	14.6 Gbp	10.5 Gbp	10.5 Gbp
Order	Synteny/genetic order^†^	Large Bins	Physical order	Physical order	Physical order
Coding genes*	133,090 HC 88,998 LC	104,091 HC 103,660 LC	107,891 HC 161,537 LC	66,559 HC 303,404 LC	67,182 HC 271,179 LC
Assembly-related resources	Archive Ensembl Plants	Archive Ensembl Plants	Ensembl Plants GrainGenes, URGI	Ensembl Plants GrainGenes	Ensembl Plants GrainGenes
	TILLING mutants		TILLING mutants		
	expVIP, wheatExp	expVIP	expVIP, eFP		
Accession	Chinese Spring	Chinese Spring	Chinese Spring	Svevo	Zavitan

^*^Number of high confidence (HC) and low confidence (LC) genes which are defined based on multiple criteria outlined in the published papers. Care must be taken when interpreting their nomenclature (see Figure 3). ^†^Chromosome arm assignment was derived from chromosome flow-sorting, while approximate intra-chromosomal ordering was established using synteny derived from grasses (GenomeZipper) and genetic mapping (POPSEQ) (Mascher et al., 2013; Mayer et al., 2014).

RefSeqv1.0 is the most widely used assembly and annotation of hexaploid wheat (available on Ensembl Plants https://plants.ensembl.org/wheat). The information from previous assemblies and annotations (Chromosome Survey Sequence (CSS) and TGACv1) are also available in the Ensembl Plants archive (https://oct2017-plants.ensembl.org) or as tracks in the Ensembl Plants genome browser interface. Ensembl Plants enables access to additional information such as SNP variation, gene trees, homoeolog assignments, and TILLING (Targeting Induced Local Lesions in Genomes) mutant information. Through this interface users can also combine knowledge from the bread, durum and wild emmer wheat genomes.

Like most of the previous hexaploid assemblies, RefSeqv1.0 is derived from the wheat landrace ‘Chinese Spring’. A combination of multiple Illumina and mate pair libraries were sequenced and assembled into scaffolds. Using a method of chromosome conformation capture called Hi-C, these scaffolds were further connected into pseudomolecules representing the 21 nuclear chromosomes of wheat, plus one additional ‘pseudo-chromosome’ (ChrU) containing all unassigned sequences (The International Wheat Genome Sequencing Consortium (IWGSC) et al., 2018).

**Figure 3.**
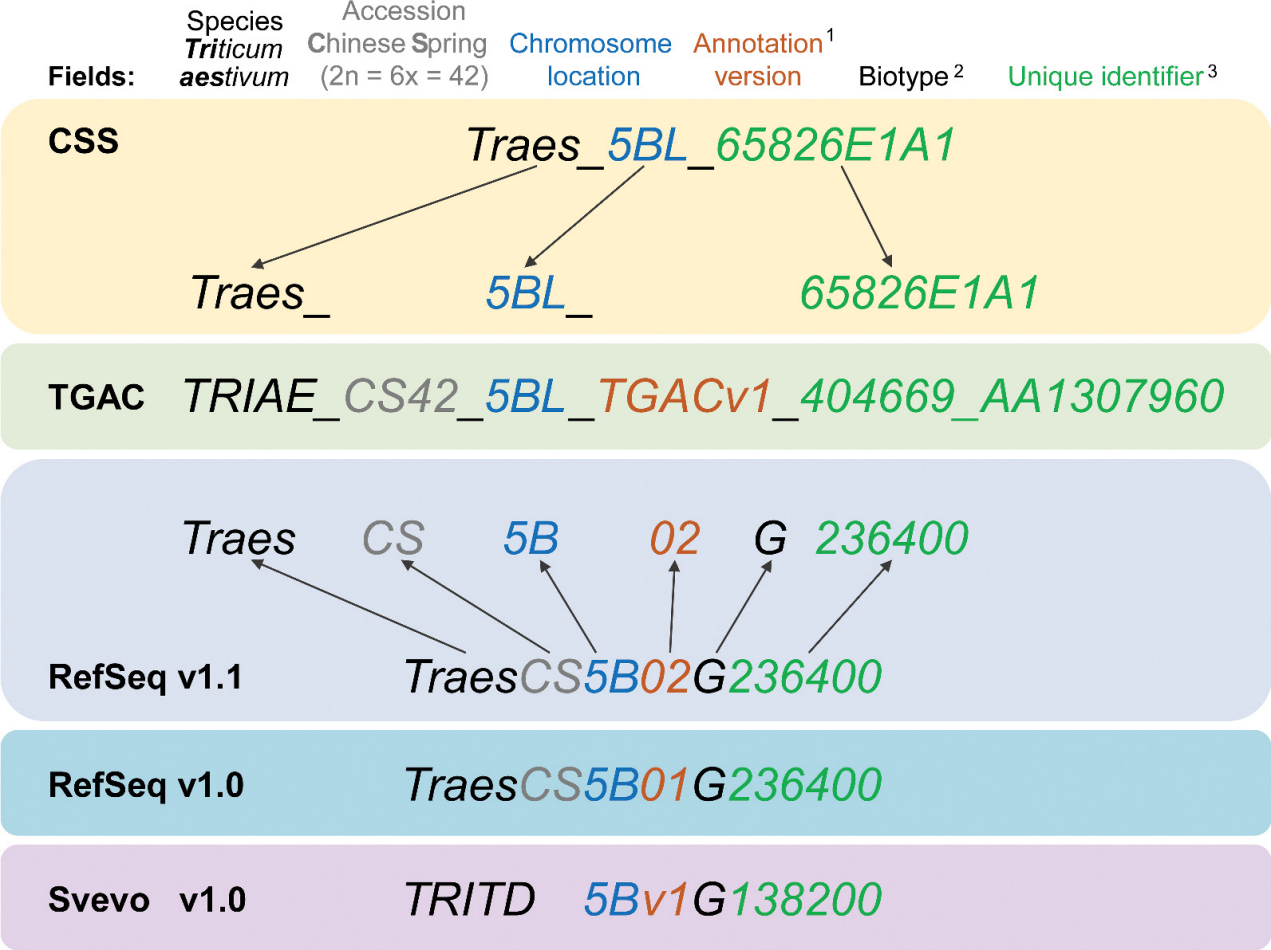
Gene model ID nomenclature description from the five available gene annotations for domesticated polyploid wheat. Here, one gene is used as an example to highlight the differences in gene ID nomenclature. Fields represented in the nomenclature are shown at the top with matching colours for the corresponding features in the gene names. Yellow background shows the CSS gene names with dark grey arrows pointing towards the corresponding field in the TGAC gene annotation (TGACv1, green background). Blue backgrounds show the gene nomenclatures for RefSeqv1.0 and v1.1 annotations (as used in Ensembl Plants), while the lilac background shows the nomenclature for Svevo v1.0 (modern durum wheat). (1) Two annotation versions are available for the RefSeqv1.0 genome assembly: RefSeqv1.0 (release annotation) and RefSeqv1.1 (improved annotation). These are differentiated by the annotation version number; ‘01’ for RefSeqv1.0 and ‘02’ for RefSeqv1.1. Otherwise, the annotations follow the same rules. (2) In the RefSeq and Svevo annotations, the biotype is represented by an additional identifier, where G = gene. (3) In the RefSeqv1.0 and v1.1 annotation, identifiers are progressive numbers in steps of 100 reflecting the relative position between gene models. For example, gene *TraesCS5B02G236400* would be adjacent to gene *TraesCS5B02G236500*. However, it is important to note that the relative positions of genes may change in future genome releases as the assembly is improved, for example, if scaffolds are rearranged. In these cases, the gene order would no longer be retained. In the gene annotation for the tetraploid durum wheat cv. Svevo, the species name is TRITD (*TRITicum Durum*) and gene identifiers increase in steps of 10, rather than by steps of 100 as in the RefSeq hexaploid wheat annotation. Note that RefSeqv1.0 and v1.1 comprises High Confidence (HC) and Low Confidence (LC) gene models. Low Confidence gene models are flagged by the ‘LC’ at the end (not shown). HC and LC genes which otherwise display the same unique identifier are **not** the same locus and are not in sequential order. Hence, *TraesCS5B02G236400* and *TraesCS5B02G236400**LC*** are both located on chromosome 5B, but are not the same gene nor are they physically adjacent. Similarly, genes from homoeologous chromosomes with the same subsequent numeric identifier are not necessarily homoeologous genes. For example, *TraesCS5A02G236400*, *TraesCS5B02G236400* and *TraesCS5D02G236400* are **not** homoeologous genes.

The gene models for the RefSeqv1.0 assembly were annotated using two prediction pipelines, which were then consolidated into a single set of gene models (RefSeqv1.0 models). A subset of these (~2,000 gene models) were later re-annotated manually, resulting in the RefSeqv1.1 gene model set (Figure 3). Over half of high confidence protein coding genes are present as exactly three homoeologous copies (1:1:1 triads), while several other combinations exist (e.g. 2:1:1 whereby there are two paralogs on the A genome, and a single homoeolog each on the B and D genomes, e.g. Gene Y in Figure 1).

The RefSeqv1.0 assembly and the RefSeqv1.1 gene models, as well as the durum and wild emmer assemblies and gene models, have been integrated into the publicly available Ensembl Plants genome browser (https://plants.ensembl.org) (Bolser et al., 2015; Howe et al., 2020). Existing variation data, both natural and induced, has been mapped to the RefSeqv1.0 hexaploid assembly and deposited in Ensembl Plants databases for visualisation via the genome browser. Integrating resources into a common reference facilitates their use and in the following sections we will discuss how to best access and utilise these resources.

## Finding wheat orthologs

Although DNA sequence homology does not equate to functional homology, it represents a good starting point for translational and/or comparative genomics. Correctly identifying orthologous genes in another plant species can be a difficult task however, especially between distantly related species like *Arabidopsis* and wheat. These two species are separated by ~200 million years of evolution and as a result both nucleotide and protein similarities are relatively low compared to more closely related species, for example, wheat and rice (*Oryza sativa* L.).

Conveniently, all the data and tools necessary for identifying putative gene orthologs from different plant species are available through the Ensembl Plants website (https://plants.ensembl.org) (Bolser et al., 2015; Howe et al., 2020). The Plant Compara pipeline has been integrated into Ensembl Plants to create ‘gene trees’ that identify and clearly display the likely orthologs of any given gene for all of the species available on its website (Vilella et al., 2009; Herrero et al., 2016). This includes the RefSeqv1.1, *Arabidopsis* TAIR10 and rice IGRSP-1.0 gene models, amongst others. This represents a quick and reliable way to identify putative wheat orthologs of a given gene (Figure 2). Tutorials for using Ensembl Plants interactively or programmatically can be found on their website or at www.wheat-training.com.

When performing a search for putative wheat orthologs via the Ensembl Plants pipeline, we would expect to find three orthologs in hexaploid wheat for most gene queries. These orthologs would normally be located on homoeologous chromosome groups, for example chromosomes 1A, 1B and 1D (Figure 1). A well-documented exception to this rule is the long arm of chromosome 4A (4AL), which has undergone translocation events with chromosome arms 5AL and 7BS (Devos et al., 1995; Ma et al., 2013). Therefore, orthologs within these translocated regions will be physically located on different chromosome groups, for example three homoeologous genes could be on chromosome arms 4AL, 5BL and 5DL. Furthermore, gene structure of wheat orthologs is often conserved with respect to rice and other closely related monocot species; this comparison can usually be done within Ensembl Plants. If this is not possible, wheat RNA-Seq data can be used to determine the gene structure. As an alternative to the Ensembl Plants Gene Trees, one can perform reciprocal protein BLAST searches to identify putative wheat orthologs. We exemplify the above-mentioned approaches, along with potential pitfalls, in more detail in the ‘Case Study’ section.

## Expression data

Determining if, when, where, and to what level a gene is expressed often constitutes one of the first steps towards its functional characterisation. Gene expression information can also be used to prioritise candidate genes underlying a quantitative trait locus (QTL) or to predict those members of a large gene family most relevant to a trait of interest. Numerous RNA-Seq datasets for wheat and many other crops have been generated and published. Although the raw data are often publicly available (e.g. via the NCBI sequence read archive, https://www.ncbi.nlm.nih.gov/sra), they are not sufficiently curated for rapid access and their use in direct comparisons is complicated due to the diversity of tissues, treatments, and origins of the samples. Expression browsers aim to centralise these public datasets and analyse them together, ideally allowing retrieval of expression information for a list of genes under different conditions. For wheat, four expression browsers are currently available: expVIP (http://www.wheat-expression.com; Borrill et al., 2016), wheat eFP browser (http://bar.utoronto.ca/efp_wheat/cgi-bin/efpWeb.cgi; Ramírez-González et al., 2018), EBI Gene Expression Atlas (https://www.ebi.ac.uk/gxa/experiments?species=triticum+aestivum), and WheatExp (https://wheat.pw.usda.gov/WheatExp; Pearce et al., 2015). Here we will focus on the first two given that they include a larger and more diverse set of samples and use the RefSeqv1.0 and v1.1 gene models described in Table 1.

Currently, expVIP includes expression data from 36 studies (1,016 RNA-Seq samples) across a diverse range of wheat tissues, developmental stages, cultivars, and environmental conditions including various abiotic and biotic stress treatments. It can display expression data for up to 250 genes at once, which can be particularly useful when working with a gene family, genes within a QTL interval, or genes involved in the same regulatory process. The expression values for each gene's homoeologs, based on the same homoeolog assignments as in Ensembl Plants, can also be displayed. The ‘homoeolog expression patterns’ of triads (genes that are present as exactly three homoeologous copies) can also be displayed as ternary plots and compared across tissues (Ramírez-González et al., 2018).

To allow comparisons across studies, the 1,016 RNA-Seq samples in expVIP were classified according to four high-level categories based on variety, tissue, developmental stage, and stress. These high-level categories are themselves divided into more detailed subcategories. These categories can be used to customise visualisation displays and allow users to select data relevant to their experimental comparisons. Data can be displayed both as transcripts per million (TPM) or as raw counts and can be directly downloaded to carry out differential gene expression analyses. Although the default gene model reference is RefSeqv1.1, users can also choose the CSS, TGACv1 and RefSeqv1.0 transcriptome references for legacy reasons. Tutorials describing expVIP are available on https://github.com/Uauy-Lab/expvip-web/wiki and www.wheat-training.com. Recently, expVIP was implemented for berry fruit species (Thole et al., 2019).

An additional resource is the electronic Fluorescent Pictograph (eFP) browser, which provides a simple visual assessment of expression data using pictures coloured according to a gene’s relative expression level. The eFP expression browser is available for several crops (e.g. potato, soybean, barley) and most recently wheat (http://bar.utoronto.ca/efp_wheat/cgi-bin/efpWeb.cgi). The wheat interface includes 209 RNA-Seq samples (also in expVIP) representing 22 tissue types from grain, root, leaf, and spike samples across multiple time points from a single hexaploid spring wheat cultivar (‘Azhurnaya’).

## Gene networks

The available RNA-Seq data provides the opportunity to identify networks of co-expressed genes. Ramírez-González et al. (2018) constructed tissue and stress-specific co-expression networks in wheat to determine whether genes from the same triad showed variable spatiotemporal expression. In addition, a GENIE3 network was developed to predict transcription factor targets across the multiple RNA-Seq samples (Huynh-Thu et al., 2010; Ramírez-González et al., 2018). Together, these networks provide a powerful set of tools for hypothesis generation using wheat-specific datasets. We have recently validated the GENIE3 network using independent RNA-Seq data from tetraploid wheat (Harrington et al., 2019). Both co-expression and GENIE3 networks are incorporated into KnetMiner (https://knetminer.org/Triticum_aestivum/).

KnetMiner is a web-application for searching and visualising genome-scale knowledge networks of, for example, *Arabidopsis*, wheat, and human diseases (Hassani-Pak et al., 2016). It aims to provide research leads for scientists who are investigating the molecular basis of complex traits. KnetMiner accepts keywords in combination with a gene list and/or genomic regions as input and searches the underlying knowledge network to identify links between these user-provided genes and keywords. A network-based visualisation, named Network View, allows users to examine complex relationships between gene networks and traits. The networks contain nodes that represent different entities such as genes, single nucleotide polymorphisms (SNPs), publications, and traits (e.g. heat or drought tolerance) that are linked via different relation types (e.g. co-expression, GENIE3-targets, protein-protein-interaction, published-in). Together, KnetMiner and the integrated gene networks provide a powerful resource for gene discovery and hypothesis generation in wheat (see Case Study below).

## Epigenomics

With the availability of the wheat genome, increasing interest has turned towards the wheat epigenome, that is heritable modifications to the genome that do not affect the DNA sequence itself, such as histone and DNA methylation. The global DNA methylome of polyploid wheat has been explored in multiple studies (Gardiner et al., 2015; Gardiner et al., 2018; Li et al., 2019). The methylome of the reference cultivar Chinese Spring was initially captured at the seedling stage (Gardiner et al., 2015), with more recent work focussing on the variation present in the seedling methylome of the 104 landraces from the Watkins core collection (Table 2; Gardiner et al., 2018). Researchers have also examined the changes in DNA methylation status as a result of biotic stress in wheat seedlings (Geng et al., 2019). The raw bisulfite sequencing data from these experiments is available through public archives, however it is not immediately accessible on genome browsers. More recently, new epigenomic data from Chinese Spring seedlings has been released which includes a wide variety of epigenetic marks such as DNA methylation, seven histone modifications, and chromatin accessibility (Li et al., 2019). This data has been made publicly available through a bespoke genome browser (http://bioinfo.sibs.ac.cn/cs_epigenome) and can be readily accessed by researchers to gain insight into the epigenomic landscape surrounding their genes of interest.

**Table 2.** Natural variation resources available in wheat.

Collection	Short description	Number of accessions	Genotyping	Data/seed availability	More information/Reference
*Wild wheat relatives and progenitor species*					
Seeds of Discovery	Wheat and wild relative accessions held by ICARDA and CIMMYT	80,000 accessions: 56,342 domesticated hexaploid (eight taxa); 18,946 domesticated tetraploid (eight taxa); 3,903 crop wild relatives included all known 27 wild species from *Aegilops-Triticum* species complex and 11 genomic constitutions.	DArT-seq	**CIMMYT Dataverse** http://hdl.handle.net/11529/10548030 **Germinate data warehouse** http://germinate.cimmyt.org/wheat. Records for all germplasm accessions can also be accessed at https://ssl.fao.org/glis/	https://seedsofdiscovery.org/
Open Wild Wheat	Accessions of *Aegilops tauschii* (D genome progenitor)	265 accessions	Whole genome shotgun sequenced (10-30x)	**Sequencing**: https://opendata.earlham.ac.uk/wheat/under_license/toronto/; **Seed**: https://www.seedstor.ac.uk/search-browseaccessions.php?idCollection=38	www.openwildwheat.org; Arora et al., 2019
Wild wheat introgression lines	Introgression lines from *Aegilops caudata, Aegilops speltoides, Amblyopyrum muticum, Thinopyrum bessarabicum, Thinopyrum elongatum, Thinopyrum intermedium, Thinopyrum ponticum, Triticum timopheevii, Triticum urartu*, rye and wheat cultivars (Chinese Spring, Higbury, Paragon, Pavon 76)	153 stable homozygous introgression lines available	35K Axiom Wheat Relative Genotyping array + 710 KASP markers (Grewal et al., 2020)	**Genotype**: https://www.nottingham.ac.uk/wrc/germplasm-resources/genotyping.aspx; **Seed**: https://www.seedstor.ac.uk/ (accessions WR0001-WR0155)	www.nottingham.ac.uk/WISP; Grewal et al., 2018a; Grewal et al., 2018b, King et al., 2018, King et al., 2017
*Synthetic hexaploid wheat*					
Synthetic hexaploid wheat	Sythetic hexaploid wheats generated using *Aegilops tauschii* (DD) + European tetraploid (AABB) wheat	50 synthetic hexaploid wheats + pre-breeding accessions; backcross populations with Robigus and Paragon also available	35K Axiom breeders array	**Genotype**: https://www.cerealsdb.uk.net/cerealgenomics/CerealsDB/axiom_download.php **Seed**: https://www.seedstor.ac.uk/ (store codes WS0001-WS0232)	https://www.niab.com/research/research-projects/designing-future-wheat
*Wheat diversity panels*					
Watkins historic collection of landrace wheats	World collection of wheat landraces grown as farmer saved seed before the 1930s. Genetically stable collection developed by two generations of single seed descent	829 accessions (core set of 119 represent majority of assayed genotypic variation). F_4:5_ mapping populations against Paragon, mainly for the core set.	35K Axiom breeders array (Allen et al., 2017); subset exome sequenced (Gardiner et al., 2018)	**Genotype**: https://www.cerealsdb.uk.net/cerealgenomics/CerealsDB/axiom_download.php **Seed**: https://www.seedstor.ac.uk/ (store codes WATDE0001-WATDE1063)	http://wisplandracepillar.jic .ac.uk/results_resources.htm ; Wingen et al., 2014; Wingen et al., 2017
GEDIFLUX (Genetic Diversity Flux) collection	Western European winter wheat varieties that individually occupied over 5% of national acreage from 1945 to 2000. Bi-parental populations with Paragon (ongoing)	479 accessions	35K Axiom breeders array	**Genotype**: https://www.cerealsdb.uk.net/cerealgenomics/CerealsDB/axiom_download.php; **Seed**: https://www.seedstor.ac.uk/ (store codes WGED0001- WGED0729)	http://wisplandracepillar.jic.ac.uk/results_resources.htm; Wingen et al., 2014
NIAB wheat association mapping panel	Bread wheat varieties released between 1916–2007. Predominantly UK varieties (68%), also other North Western European countries e.g. France (10%) and Germany (8%)	480 accessions	90K SNP array	**Seed, Genotype and Pedigree**: https://www.niab.com/research/research-projects/resources	Fradgley et al., 2019
OzWheat diversity panel	Genetic diversity in Australian wheat breeding (colonial landraces 1860s, first Australian-bred cultivars 1890s, CIMMYT-derived semi dwarfs 1960s, post 2000 wheat)	285 accessions	90K SNP array + additional 26K SNPs from transcriptome data	**Seed and Genotype**: contact Shannon Dillon from CSIRO (Shannon.Dillon@csiro.au)	
Vavilov wheat collection	Hexaploid wheat accessions including landraces, historic breeding lines and cultivars. Pure lines generated by single seed descent	295 accessions	DArtT-seq (34,311 polymorphic markers)	**Genotype**: Lee Hickey at The University of Queensland (l.hickey@uq.edu.au) **Seed**: Australian Grains Genebank (sally.norton@ecodev.vic.gov.au)	Riaz et al., 2017
WHEALBI wheat panel	Worldwide wheat accessions including diploid and tetraploid wild relatives, old hexaploid landraces and modern elite cultivars	487 accessions	Exome capture (~600,000 genetic variants in ~40,000 genes; 12,000 genes identified as putative presence/absence variation compared to RefSeqv1.0)	**Genotype**:https://urgi.versailles.inra.fr/download/iwgsc/IWGSC_RefSeq_Annotations/v1.0/iwgsc_refseqv1.0_Whealbi_GWAS.zip; **Seed**: https://www.gbif.org/dataset/a52ca10a-136a-4072-a6de-3ec6e7852365	Pont et al., 2019
Global Durum Wheat (GDP) panel	Diversity used in durum wheat breeding programs globally, including landraces and modern varieties	1,056 accessions	90K SNP array	**Genotype**: ms in preparation; **Seed**: ICARDA genebank http://indms.icarda.org Filippo Bassi, F.Bassi@cgiar.org	
Tetraploid wheat Global Collection (TGC)	Wild emmer wheat, domesticated emmer, durum wheat landraces and other tetraploid wheat sub-species (*Triticum aethiopicum, Triticum carthlicum, Triticum polonicum, Triticum turanicum, Triticum turgidum, Triticum karamyschevii* and *Triticum petropavlovsky*)	1,856 accessions	90K SNP array	**Genotype:** GrainGenes; **Seed:** on request for non-commercial use from University of Bologna (marco.maccaferri@unibo.it and roberto.tuberosa@unibo.it)	Maccaferri et al., 2019
*MAGIC populations*					
CSIRO, Aus	4-way (parents Baxter, Chara, Westonia, Yitpi); 8-way (parents Baxter, Westonia, Yitpi, AC Barrie (Canada), Xiaoya54 (China), Volcani (Israel), Pastor (Mexico), Alsen (USA))	1,500 (4-way) and 3,000 (8-way) RILs	90K SNP array, microsatellite and DArT markers > 20,000 SNPs mapped in each population	**Seed and Genotype**: on request from CSIRO (Bill.Bovill@csiro.au)	Huang et al., 2012; Shah et al., 2019
NIAB, UK	8-way (parents Alchemy, Brompton, Claire, Hereward, Rialto, Robigus, Xi19, Soissions); 16-way (Banco, Bersee, Brigadier, Copain, Cordiale, Flamingo, Gladiator, Holdfast, Kloka, Maris Fundin, Robigus, Slejpner, Soissons, Spark, Steadfast, Stetson)	NIAB 8-way MAGIC:>1,000 RILs; NIAB 16-way MAGIC: ~600 RILs	35K Axiom breeders array. Genome sequence (Claire, Robigus, others underway). Exome capture sequence of 16-way parents. Skim-seq of all RILs underway.	**Claire and Robigus genomes**: https://opendata.earlham.ac.uk/opendata/data/Triticum_aestivum/EI/v1.1/ **Genotyping and Seed**: https://www.niab.com/research/research-projects/resources	Mackay et al., 2014; Gardner et al., 2016
Germany	8-way (Event, Format, BAYP4535, Potenzial, Ambition, Bussard, Firl3565, Julius)	394 F_6:8_ RILs	5,435 SNPs from SNP array	**Genotype and pedigree**: http://doi.org/10.14459/2018mp1435172 (click the ‘open attachment browser’ link); **Seed**: Bavarian State Research Centre for Agriculture (Freising, Germany)	Stadlmeier et al., 2018
Germany	WM-800, 8-way (Patras, Meister, Linus, JB Asano, Tobak, Bernstein, Safari, Julius)	910 F_4:6_ RILs	15K Infinium iSelect SNP array	**Genotype and pedigree:** https://www.ncbi.nlm.nih.gov/pmc/articles/PMC6069784; **Seed:** on request from Martin Luther University, Germany (klaus.pillen@landw.uni-halle.de)	Sannemann et al., 2018
Durum	4-way (Claudio (Italy), Colosseo (Italy), Neodur (France), Rascon/2*Tarro (advanced CIMMYT line))	334 F_7:8_ RILs	90K SNP array	**Genotype and pedigree:** https://onlinelibrary.wiley.com/doi/full/10.1111/pbi.12424; **Seed:** on request for non-commercial use from University of Bologna (marco.maccaferri@unibo.it and roberto.tuberosa@unibo.it)	Milner et al., 2016

## Functional studies

After identifying a gene of interest there are now several options and resources available for functional characterisation and validation in wheat (Figure 2). These include resources based both on natural and induced variation and can involve both transgenic and non-transgenic approaches. It is important to remember that due to the polyploid nature of wheat, there is often functional redundancy between homoeologs (Borrill et al., 2015). This means that it may be necessary to manipulate all homoeologs and paralogs simultaneously to measure a strong phenotypic effect (see the ‘Strategies for Use’ section below for more information).

### Induced variation

#### TILLING

Polyploid species, such as wheat, are well suited to mutational approaches as the functional redundancy in their genomes allows for the tolerance of a higher mutational load compared with diploid species (Tsai et al., 2013; Uauy et al., 2017). Bespoke mutant populations can be developed and screened for desired mutations in a gene of interest, though the screening process is arduous and time-consuming. To overcome this barrier, an *in silico* wheat TILLING resource has been developed (Krasileva et al., 2017). This resource consists of two ethyl methanesulphonate (EMS) mutagenised populations: 1,535 lines of the tetraploid durum wheat variety ‘Kronos’ and 1,200 lines of the hexaploid bread wheat variety ‘Cadenza’. Exome capture and Illumina sequencing of these 2,735 mutant lines was then carried out. The raw data was originally aligned to the CSS reference, mutations were identified, and their effects predicted based on the CSS gene models (Krasileva et al., 2017). Alleles predicted *in silico* to be deleterious (e.g. premature stop codons, splice site mutations, non-synonymous amino acid substitutions with SIFT score <0.05), were identified for ~90% of the captured wheat genes (Krasileva et al., 2017), thus making this a powerful resource for rapidly identifying mutations in a gene of interest (Figure 2). The raw data has now been aligned to the RefSeqv1.0 genome, allowing mutation identification and effect prediction based on the RefSeqv1.1 gene models. These updated data are publicly available on Ensembl Plants (see Case Study for details). For legacy purposes, the mutations called against the CSS reference remain available via www.wheat-tilling.com. However, caution should be exercised as the mutation effects here are predicted based on the CSS gene models, which are known to be less reliable than the RefSeq gene models (Brinton et al., 2018).

There are several important considerations when selecting a mutant line for characterisation. First, it is essential to check the predicted effect of mutations in the context of a complete and experimentally validated gene model. Second, in most cases, crossing is necessary to combine mutations in homoeologous genes in order to generate a complete null individual. Third, mutant lines will contain a high level of background mutations: a typical mutant line has between 50 (tetraploid) and 110 (hexaploid) mutations predicted to result in a truncated protein. Depending on the phenotype of interest (i.e. qualitative vs. quantitative) several rounds of backcrossing may be required before the phenotype can be assessed (see ‘Strategies for Use’). Lastly, if the gene of interest is missing or is already a null allele in Kronos or Cadenza (which can be determined using the full genome sequences of the two cultivars), mutant populations of other genotypes are available (e.g. Dong et al., 2009; Chen et al., 2012; Bovina et al., 2014; Sestili et al., 2015; Colasuonno et al., 2016), although these would need to be screened using conventional PCR-based approaches. Additional practical information about selecting mutant lines and downstream analyses can be found at www.wheat-training.com/functional-studies and in Uauy et al. (2017).

#### Transgenic approaches

Stable transformation of wheat is possible and can be performed using a variety of methods including both particle bombardment (Vasil et al., 1992; Sparks and Jones, 2009) and *Agrobacterium*-mediated transformation (Cheng et al., 1997; Sparks et al., 2014). Generating stable transgenic lines in wheat most commonly involves transforming immature wheat embryos and subsequent callus regeneration (Harwood, 2012). Reports in the literature of *Agrobacterium*-mediated wheat transformation generally describe low transformation efficiencies with average efficiencies of around 5%. An efficient, but patented, transformation system is available through licence from Japan Tobacco (www.jti.co.jp). Transformation by overexpression of transcription factors such as maize *Baby Boom* and *Wuschel2* has also yielded improved transformation efficiencies in monocots (Lowe et al., 2016), although there are no formal reports yet in wheat. Recently, an open-access wheat transformation system with transformation efficiencies of up to 25% was published (Hayta et al., 2019), albeit for a single cultivar.

Using transgenic approaches, gene expression can be altered in a variety of ways such as overexpressing or ectopically expressing the gene of interest using either constitutive, tissue-specific or inducible promoters (Hensel et al., 2011). Similarly, RNA-interference (RNAi) has been used successfully in wheat to reduce gene expression with the added benefit that constructs can be designed to target all homoeologous genes simultaneously, thereby overcoming the potential drawback of functional redundancy among homoeologs (Fu et al., 2007). In addition to altering expression patterns, modified proteins can also be introduced (e.g. including tags) for downstream experiments such as ChIP-Seq (Deng et al., 2015) or localisation studies (Harwood et al., 2005). However, these are still not commonly employed in wheat research. As transformation methods have only been optimised for a limited number of wheat varieties (e.g. Richardson et al., 2014), it is important to understand whether the gene is expressed/functional in the chosen variety when defining transgenic strategies (see ‘Strategies for Use’).

Recent developments in genome editing technologies provide new opportunities for manipulating genes in wheat. TALEN and CRISPR/Cas9-mediated genome editing has been successfully demonstrated in wheat both in transient expression systems (Shan et al., 2014) and stably transformed plants (Wang et al., 2014b; Luo et al., 2019), using a range of methods reviewed in Uauy et al. (2017). Currently, most studies have introduced specific point mutations or small deletions leading to subsequent protein disruption, although the technology holds the potential for complex applications such as allele swapping or gene insertion, as reviewed by Puchta (2017). Similar to RNAi, constructs for Cas9-mediated gene editing can be designed to target all homoeologs simultaneously (Zhang et al., 2016; Howells et al., 2018). Due to the current efficiency of genome editing however, the likelihood of obtaining mutations in all homoeologs in a single T_0_ plant remains low (0.9%; Zhang et al., 2016) and subsequent crosses to combine multiple edited targets are likely to be required.

A major limitation of using transgenic approaches to manipulate agronomically relevant traits is the associated legal and regulatory constraints. To overcome these, the nuclease transgene can be segregated away from the edited gene(s) in subsequent generations. However, in Europe, and in contrast to many other countries in the world, the resulting plants would be regulated as transgenics due to the 2018 ruling on genome editing by the European Court of Justice (ECJ). Some studies have documented CRISPR/Cas9-editing in wheat without transgene integration, for example, by delivering the CRISPR/Cas9 components as ribonucleoproteins (RNPs). As no foreign DNA is used in CRISPR/Cas9 RNP-mediated genome editing, the wheat mutants obtained are completely transgene free (Liang et al., 2017), although still not exempt from the ECJ regulation.

#### Virus-Induced Gene Silencing

Virus-Induced Gene Silencing (VIGS) involves transient knock-down of expression of target genes followed by assessment of the resulting phenotype (Lee et al., 2012). The most widely used vectors for VIGS in wheat are those derived from barley stripe mosaic virus (BSMV), a plant virus with a tripartite RNA genome that readily spreads throughout tissues following mechanical rub-inoculation onto the leaves. All three BSMV genomic RNAs, RNAα, RNAβ and RNAγ, are required to cause infection. RNAγ has been modified to allow insertion of short (up to 350 bp) plant mRNA derived sequences. Infection of plants with the resulting recombinant virus induces a natural post-transcriptional gene silencing defence mechanism that targets the viral RNA, but also the endogenous plant mRNA having high level (>70%) nucleotide identity with the plant sequence inserted into RNAγ, for degradation. A detailed protocol for VIGS is available at www.wheat-training.com (Figure 2).

VIGS in wheat has been used primarily to investigate disease resistance in a range of varieties, and has been restricted to a few tissue types such as leaf (Lee et al., 2015), young seedlings (Zhang et al., 2017a) and spikes (Ma et al., 2012). However, in principle, BSMV-mediated VIGS can be applied to any wheat genotype and to almost any gene of interest. This functional genomics tool is particularly useful when analysing multiple candidate genes, for example in map-based cloning projects (i.e. when physical intervals contain several candidate genes), or from RNA-Seq differentially expressed datasets. VIGS is also useful in wheat genotypes that are difficult to transform and in those for which mutant/TILLING populations are unavailable. VIGS can be used for simultaneous silencing of all homoeologs or, in principle, entire small gene families without the need for further genetic crosses.

### Natural Variation

Although using induced variation presents a clear route to understand the function of specific genes in wheat, the wealth of natural variation in wheat lines, and populations based on this variation, presents an alternative route to discover genes and correlate them with function. For example, populations differing for alleles of the gene of interest could be used to rapidly infer the role of the gene. In order to capture the diversity within wheat and create populations to test gene function, natural variation has been extensively documented. Most studies have focused on SNPs between varieties that can be quickly assayed through SNP arrays designed from gene coding sequences and untranslated regions (UTRs) (Wang et al., 2014a; Winfield et al., 2016; Allen et al., 2017), described in Borrill et al. (2015) and www.wheat-training.com. Thousands of varieties and landraces have been processed using these arrays and datasets are available through websites such as TCAP (https://triticeaetoolbox.org/wheat) (Blake et al., 2016) and CerealsDB (http://www.cerealsdb.uk.net/cerealgenomics/CerealsDB) (Wilkinson et al., 2016). Given that all SNPs from the latter have been incorporated into Ensembl Plants, this means that large *in silico* allelic series are readily available for many genes of interest.

Beyond SNP variation, two recent studies (He et al., 2019; Pont et al., 2019) applied exome capture to diverse wheat lines to characterise the natural variation throughout the coding regions of wheat. These studies identified millions of SNPs within coding sequences in over 1,000 wheat lines, including hexaploid cultivars and landraces, and tetraploid and diploid relatives. The data (available at http://wheatgenomics.plantpath.ksu.edu/1000EC and https://urgi.versailles.inra.fr) will allow rapid characterisation of the extent of variation within genes of interest. These changes in coding sequences may have direct phenotypic consequences, however the impact of most of these variants remains unknown.

Therefore, despite this wealth of data, the challenge remains to define the functional significance of this variation. Traditionally, mapping populations or association panels would need to be developed or assembled, and then genotyped, to assess how particular SNPs or haplotypes affect the trait of interest. In wheat, many of these resources are now publicly available (Figure 2), thus facilitating the functional characterisation of genes of interest. We describe some of these resources below and include links to access genotypes, sequences and seeds in Table 2. Further details are available at www.wheat-training.com.

#### Wild wheat relatives and progenitor species

There is relatively low genetic variation in elite bread wheat varieties, especially on the D genome. This typically reflects adaptation and selection from landraces over a long time period, combined with the genetic bottleneck effects associated with the rare natural hybridisation events between the diploid and tetraploid ancestral wheat species that led to the evolution of hexaploid wheat. Wheat is related to several other grass species, many of which are wild and uncultivated. These wild relatives provide a vast and largely untapped reservoir of genetic variation for many agronomically important traits. A wealth of cytogenetic stocks for these wild relatives have been created over the last 100 years by researchers globally, reviewed by Mujeeb-Kazi et al. (2013). The recent genotyping and sequencing of some of these resources makes them especially suitable for gene functional characterisation (Table 2).

#### Synthetic hexaploid wheat

Another approach to capture variation in wheat progenitors is via ‘re-synthesis’, the process used to create synthetic hexaploid wheat (SHW). SHWs are typically created by crossing tetraploid durum wheat with the diploid D-genome progenitor *Aegilops tauschii.* Approximately 400 SHWs were developed at CIMMYT in Mexico during the 1990s (Mujeeb-Kazi et al., 1996) and these have been extensively utilised in CIMMYT and international wheat breeding programmes (e.g. Gororo et al., 2002; Ogbonnaya et al., 2007). More recently, NIAB (UK) have developed a new SHW resource encompassing 50 SHWs along with pre-breeding derivatives. This germplasm, alongside marker data, is publicly available (Table 2).

#### Wheat diversity panels

Numerous collections of wheat landraces, varieties and breeders’ lines are available from research centres around the world. These panels represent valuable sources of potential genetic variation for targeted exploitation within wheat research and pre-breeding pipelines, especially when associated with existing genotypic and phenotypic datasets (Table 2). Further details are available at www.wheat-training.com.

#### Multiparent Advanced Generation Inter-Cross (MAGIC) populations

MAGIC populations have been developed for many crop species (Huang et al., 2015; Cockram and Mackay, 2018). The multiple generations of inter-crossing required to create MAGIC populations results in highly recombined chromosomes, which enables the use of approaches such as genome wide association scans (GWAS) and whole-genome average interval mapping (WGAIM; Verbyla et al., 2007) to define small genetic intervals for traits of interest as reviewed by Verbyla et al. (2014). Likewise, the use of multiple parents allows more allelic variation to be examined compared to typical bi-parental populations (Cockram and Mackay, 2018). In wheat, seven MAGIC populations are currently publicly available which are constructed from 4, 8 or 16 founders. Parent information and further details can be found in Table 2.

### Combining induced and natural variation for a holistic picture of gene function

To date, natural variation has largely been used for forward genetics approaches such as mapping genetic regions underlying a phenotypic trait of interest. However, there is now an opportunity to apply natural variation in wheat for reverse genetics studies to complement transgenic, gene editing and induced variation approaches. For example, the pre-harvest sprouting locus *Phs-A1* was reported by two independent studies to be underpinned by different genes: in one case by a pair of tandem duplicated *Plasma Membrane 19* (*PM19-A1* and *PM19-A2*) genes (Barrero et al., 2015), and in the other by a *mitogen-activated protein kinase kinase 3* (*TaMKK3-A*) gene (Torada et al., 2016). Transgenic approaches seemed to validate the role of both *PM19* and *TaMKK3-A* to influence pre-harvest sprouting. However, by using eleven bi-parental populations and a MAGIC population segregating for the *Phs-A1* locus, it was possible to break the linkage with the polymorphism in *PM19* and confirm that the causal gene in all populations was *TaMKK3-A* (Shorinola et al., 2017). This example illustrates the power of natural variation to validate the causal variants underpinning phenotypes in wheat.

Populations exploiting natural variation can also be used to validate gene function. For example, *TEOSINTE BRANCHED1* (*TB1*) was identified as a regulator of wheat spike architecture using a 4-parent Australian MAGIC population, and this function was confirmed using induced variation (TILLING and transgenic overexpression) and natural variation in the 8-parent UK MAGIC population (Dixon et al., 2018). Interestingly, whilst *TB1* was important in both MAGIC populations, different homoeologs underpinned the variation: *TB1-D1* in the Australian population and *TB1-B1* in the UK population. This study suggests that by using natural variation, we can start to understand the nuanced regulation of phenotypes in wheat elicited by individual homoeologs. Together, these examples show that researchers now have at their disposal a powerful toolkit to combine induced and natural variation to study gene function in wheat.

### Moving towards a wheat pangenome

Increases in DNA sequencing outputs and related technologies have allowed the assembly of chromosome scale assemblies for multiple cultivars in major crops such as maize (https://nam-genomes.org/), rice (Zhou et al., 2019) or oilseed rape (Song et al., 2020). For wheat, 16 hexaploid (eight with spring habit, and eight with winter habit), and three tetraploid varieties/accessions have been assembled, several to a similar standard as the reference Chinese Spring genome (Table 3). Annotation of most of these varieties is ongoing through the 10+ Wheat Genomes Project (http://www.10wheatgenomes.com) and will provide information on the core (genes shared by all assembled varieties) and dispensable genes (genes shared among a few varieties). In addition, presence absence variation, copy number variation, structural rearrangements (inversions/translocations), and variation across non-coding regions are being quantified. Importantly, several of these genotypes are part of the resources outlined above, for example sequenced TILLING populations (Kronos and Cadenza). These assemblies will be integrated into Ensembl Plants and are available for download under Toronto Agreement (https://wheat.ipk-gatersleben.de/).

**Table 3.** Tetraploid and hexaploid wheat genome assemblies that are currently available, in addition to the Chinese Spring reference hexaploid genome.

Variety	Habit	Origin	Availability †
*Hexaploid wheat*			
CDC Landmark	spring	Canada	10+ Genome Project
CDC Stanley	spring	Canada	10+ Genome Project
Paragon	spring	UK	10+ Genome Project
Cadenza	spring	UK	10+ Genome Project
LongReach Lancer	spring	Australia	10+ Genome Project
Mace	spring	Australia	10+ Genome Project
Synthetic W7984	spring	Mexico	Chapman et al. (2015)
Weebill	spring	Mexico	10+ Genome Project
Arina*LrFor*	winter	Switzerland	10+ Genome Project
Julius	winter	Germany	10+ Genome Project
Jagger	winter	US	10+ Genome Project
Robigus	winter	UK	10+ Genome Project
Claire	winter	UK	10+ Genome Project
Norin61	winter	Japan	10+ Genome Project
SY Mattis	winter	France	10+ Genome Project
Spelt (PI190962)	winter	Europe	10+ Genome Project
*Tetraploid wheat*			
Zavitan*	-	Israel	Avni et al. (2017)
Svevo	spring	Italy	Maccaferri et al., 2019
Kronos	spring	US	10+ Genome Project

^*^‘Zavitan’ is a tetraploid wild emmer (*T. dicoccoides*) accession. ^†^Varieties included within the 10+ Wheat Genomes Project can be accessed through the Earlham Grassroot Genomics portal (https://wheatis.tgac.ac.uk/grassroots-portal/blast) and the 10+ Wheat Genomes project portal (http://webblast.ipk-gatersleben.de/wheat_ten_genomes) (subset of varieties in each). The ‘Svevo’ genome can be accessed through https://www.interomics.eu/durum-wheat-genome and Ensembl Plants. ‘Synthetic W7984’ and ‘Zavitan’ can be accessed through the Grassroot Genomics, and Ensembl Plants, respectively.

## Strategies for use

### Variety selection and growth conditions

Whilst resources are now available for the functional validation of target genes in wheat, practical knowledge is also required to maximise the value of these resources. Firstly, wheat varieties are adapted to different growing conditions (e.g. daylength and vernalisation requirements) making it important to consider the conditions under which functional validation will be conducted. If phenotyping will be undertaken in greenhouse or controlled environment conditions then most varieties will be suitable, although varieties without vernalisation requirements are faster to grow (details on wheat growth conditions at www.wheat-training.com). If field trials are required for phenotypic characterisation (e.g. yield-related traits), local adaptation is often necessary for correct interpretation of results given genotype x environment interactions. For example, the sequenced TILLING populations (Kronos and Cadenza) do not require vernalisation, facilitating greenhouse experiments, and originate from different regions of the world, allowing field trials under different environments (Kronos is a Californian variety adapted to warm dry weather whereas Cadenza is a UK variety adapted to cooler conditions).

For CRISPR/Cas9 and other non-transient transgenic approaches several varieties may be used, although relatively few wheat varieties have been shown to display high enough transformation efficiencies to be practical. This means that traditionally most transgenic studies in wheat have been limited to a few varieties, such as ‘Fielder’, Cadenza, ‘Bobwhite’, ‘Kenong 199’ and Kronos (Li et al., 2012; Richardson et al., 2014; Liang et al., 2017; Hayta et al., 2019). This is now changing thanks to work by groups at NIAB (UK), CAAS (China) and CSIRO (Australia) who have successfully transformed 39 (Wallington, 2015), 15 (Wang et al., 2017) and six (Richardson et al., 2014) varieties, respectively. However, the *Agrobacterium*-mediated transformation efficiencies in all these studies still differ between varieties. Correct varietal selection for transformation is critical for functional studies, given that some varieties might not be suitable to study a particular phenotype (e.g. if the variety is resistant to a disease and hence cannot be used to test a candidate resistance gene). Similarly, it is important to assess whether the gene of interest is present/functional in the chosen variety, for example through PCR amplification and sequencing of the gene. For several varieties this can now be done quickly by direct examination of their genome sequence (Table 3).

### Combining mutations for complete knock-outs in polyploid wheat

**Figure 4.**
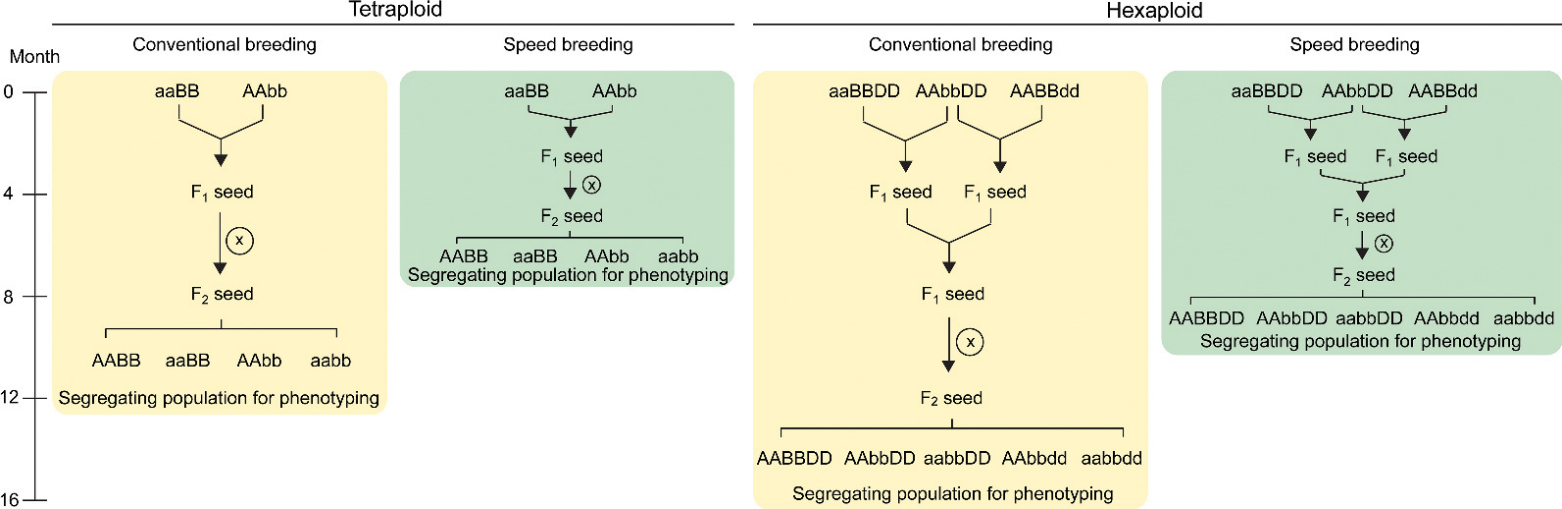
Crossing scheme to combine TILLING or CRISPR/Cas9 single mutants in wheat. In tetraploid wheat, mutations in the A and B genome homoeologs can be combined through a single cross. The F_1_ plants are self-pollinated to produce a segregating F_2_ population which contains homozygous double and single mutants, as well as wild type plants (screening using molecular markers required; only four genotypes shown). These F_2_ progeny can be characterised for the phenotype of interest. The use of ‘speed breeding’ (Watson et al., 2018), reduces the time taken to reach this phenotyping stage from 12 (yellow) to 7.5 months (green). In hexaploid wheat, a second round of crossing is required to combine the mutant alleles from all three homoeologs. The F_2_ progeny segregating for the three mutant alleles can be genotyped using molecular markers to select the required combination of mutant alleles (only five genotypes shown; all factorial combinations are possible). Speed breeding reduces the time taken to generate triple homozygous mutants for phenotyping to 10 months (green), compared to 16 months in conventional conditions (yellow). Self-pollination is represented by an X inside a circle. Combinations of wild type alleles from the A (AA), B (BB) and D (DD) genomes, as well as the mutant alleles from each genome (aa, bb and dd, respectively) are indicated.

As we noted earlier, the polyploid nature of wheat means that it normally has multiple homoeologous copies of every gene. These copies typically have highly similar coding DNA sequence and may have redundant functions (Borrill et al., 2015). Therefore, to characterise the function of a gene in wheat it is often necessary to knock out all three homoeologs. This may be achieved by simultaneously targeting all three copies using either RNAi (e.g. Uauy et al., 2006) or CRISPR/Cas9 (e.g. Zhang et al., 2017b). A large number of transformants need to be screened to identify a null in all three genomes from a CRISPR construct (Zhang et al., 2017b; Howells et al., 2018). If the targets are more divergent it may not even be possible to use a single guide RNA to target all three homoeologs, in which case several guides may be used through multiplexing. Alternatively, separate knock-outs for each homoeolog can be generated by CRISPR/Cas9 or identified in TILLING populations. The mutations in each homoeolog can be combined by crossing (for details see www.wheat-training.com), with two crosses necessary to combine knock-out mutations in each of the three homoeologs in hexaploid wheat (Figure 4). Tetraploid wheat, with only two homoeologs, can be used to accelerate functional characterisation as it requires just one cross to create complete knock-out mutants (Figure 4). After self-pollination of this F_1_, phenotyping of the trait of interest can be initiated in the F_2_ generation by comparing homozygous double knock-out mutants to the sibling wild type plants. It is important to note that TILLING lines contain many background mutations and backcrossing may be required to overcome the confounding effects of background mutations on target phenotype. More details on these strategies are published in Uauy et al. (2017).

### Accelerating crossing, generation time, and phenotyping

The need to combine multiple mutations/alleles and carry out backcrossing to remove background mutations takes a considerable amount of time, with at least four months required per generation in a spring wheat genetic background. Recently, the ‘speed breeding’ technique has been implemented in wheat (and other crops such as barley, canola and chickpea), which uses extended day lengths of 22 hr and improved light quality to accelerate the generation time in wheat (Ghosh et al., 2018; Watson et al., 2018). Reduction of generation times to 8–10 weeks is achieved through an accelerated growth rate and harvesting of immature seeds 2–3 weeks post anthesis. The immature seeds are dried and then imbibed in the cold, resulting in nearly 100% germination. Incorporating speed breeding within crossing programmes can reduce the time required to produce and phenotype double mutants in tetraploid wheat to less than 7.5 months and triple mutants in hexaploid wheat to less than 10 months (Figure 4). In addition to reducing generation times, it has been shown that several traits of interest such as disease resistance, height and flowering time can be properly characterised under speed breeding conditions (Watson et al., 2018).

### Homoeolog-specific PCR markers

To carry out the crossing schemes described above, it is essential to be able to select for the mutations of interest. In polyploid wheat it is necessary to track mutations in each homoeolog separately, which can be achieved using homoeolog-specific genetic markers. Primers can be designed to include a homoeolog-specific SNP at the 3’ end of the primer. The primer will amplify the targeted homoeolog more efficiently than the non-targeted homoeolog(s) resulting in genome-specific amplification. Rapid design of homoeolog-specific primers can be achieved using the PolyMarker pipeline (Ramirez-Gonzalez et al., 2015) and webserver (http://www.polymarker.info/). Routinely, genotyping of SNPs is carried out using Kompetitive Allele Specific PCR (KASP) markers which are relatively high throughput, inexpensive and can be used in individual lab settings equipped with PCR machines and widely available fluorescence plate readers (Allen et al., 2011). The SNP to be genotyped (e.g. between mutant and wild type) is located at the 3’ end of the two alternative allele-specific primers used in the KASP reaction (one for the mutant and one for the wild type allele), whilst the homoeolog-specific SNP is located at the 3’ end of the common primer. Amplification should thus be both homoeolog-specific and allele-specific. Further guidance on the design of genome-specific primers and KASP markers is available at www.wheat-training.com.

## Case study

**Figure 5.**
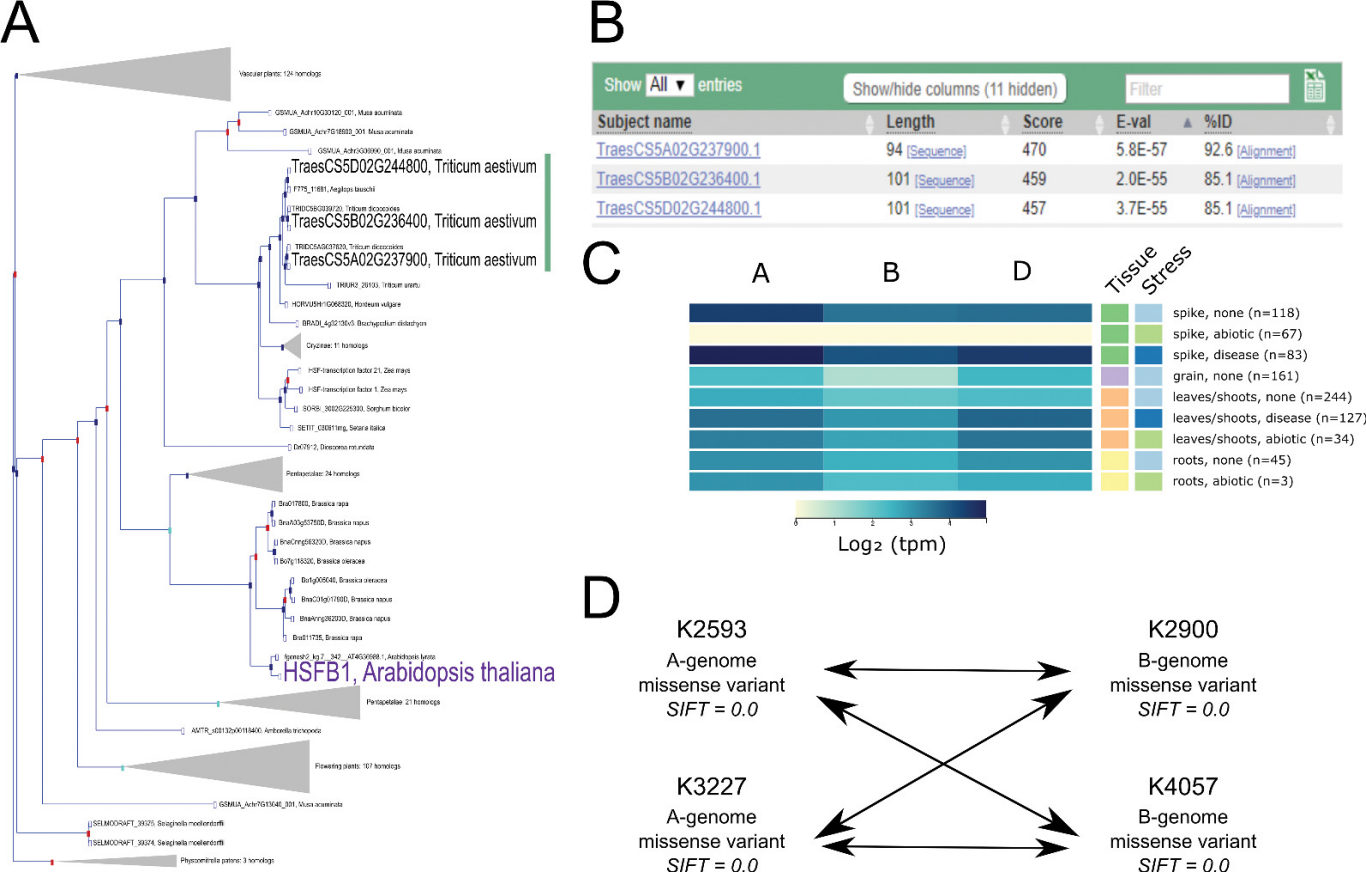
Case study exemplifying use of available resources for gene functional characterisation in wheat. (**A**). The Ensembl Plants Gene Tree illustrates the identification of the wheat triad (green bar) most closely related to *AtHSFB1* (shown in purple). (**B**) Using *Os09g0456800* (the rice ortholog of *AtHSFB1*) as a BLASTp query against wheat predicted proteins independently identifies the same wheat triad. (**C**) Examination of RNA expression data from www.wheat-expression.com shows that the wheat triad is most highly expressed in the spike, with differential expression in abiotic and disease stress conditions. The samples are identified by tissue of origin (spike, green; grain, purple; leaves/shoots, orange; roots, yellow) and stress (none, light blue; abiotic, green; disease, dark blue) as they are on the website. (**D**) After identification of suitable wheat TILLING mutants, A and B genome homoeologs are combined via this example crossing scheme, demonstrating the four crosses required between the two selected mutations in each homoeolog. Note that the functional validation proposed in (**D**) is carried out using the tetraploid mutant population.

To put the previous resources into context, we present a case study for obtaining wheat mutants and expression data using a gene of interest from *Arabidopsis thaliana*. The heat shock factor-like transcription factor *TBF1*, also known as *HsfB1*, is a critical regulator of the plant growth-to-defence transition (Pajerowska-Mukhtar et al., 2012), and the response to heat stress (Guo et al., 2016). We therefore hypothesise that its wheat orthologs may have a similar role in regulating defence and/or abiotic stress responses (Ikeda et al., 2011). The first step to test this hypothesis is to identify wheat *TBF1* orthologs, which can be done using the Ensembl Plants Gene Tree (Bolser et al., 2015), which displays predicted orthologs for all species included in Ensembl Plants. *TBF1* is one of five *HSFB* orthologs, named *HSFB1, 2A, 2B, 4,* and *5*, respectively. Examination of the Ensembl Plants Gene Tree shows a single wheat triad that falls within the *HSFB1* clade, located on the group five chromosomes (Figure 5A). It is important to note that most gene models were annotated in an automated manner and hence gene structures are likely to contain some errors, pending manual curation. We would thus recommend that researchers manually inspect the annotation of their genes of interest before proceeding further with their analyses.

To support the predicted *Arabidopsis*-wheat orthologs obtained from Ensembl Plants, we recommend carrying out comparisons between wheat and rice to establish orthology between these cereal species. Both the wheat homoeologs and the rice gene model *Os09g0456800* have the same gene structure, consisting of two exons with a conserved intron/exon boundary position. To further support the relationship of the rice gene to the wheat homoeologs, the predicted rice protein can be used as a query for BLASTp analysis of the wheat proteome in Ensembl Plants; the expected wheat orthologs are the top three hits for the A, B, and D genomes (Figure 5B).

Having identified the wheat orthologs of *Arabidopsis TBF1*, we can examine and compare expression profiles using the expVIP browser (www.wheat-expression.com) (Borrill et al., 2016; Ramírez-González et al., 2018; Figure 5C). All three wheat homoeologs have similar expression profiles, with expression changes in the spike under disease and abiotic stress. This is consistent with the eFP browser data which shows high expression in the spikelet and awns of the non-stressed plants, as well as in more mature leaf tissues (Winter et al., 2007; Ramírez-González et al., 2018). The expression data suggests that the wheat *TBF1* homoeologs are most strongly expressed in the spike and may have differential expression in response to biotic and abiotic stress. We can also explore the epigenetic environment of the three homoeologs using the bread wheat epigenomic map (http://bioinfo.sibs.ac.cn/cs_epigenome; Li et al., 2019). A large peak for the H3K9ac histone modification at the 5′ end of the homoeologs is indicative of active transcription from the promoter, corresponding with the observed gene expression. In contrast, the A-homoeolog *TraesCS5A02G237900* is flanked by two genes which have low expression at the seedling stage, and correspondingly low levels of H3K9ac modifications in their promoters. It is worth noting that the epigenomic browser uses RefSeqv1.0 gene models, rather than the RefSeqv1.1 gene models used on Ensembl Plants.

**Figure 6.**
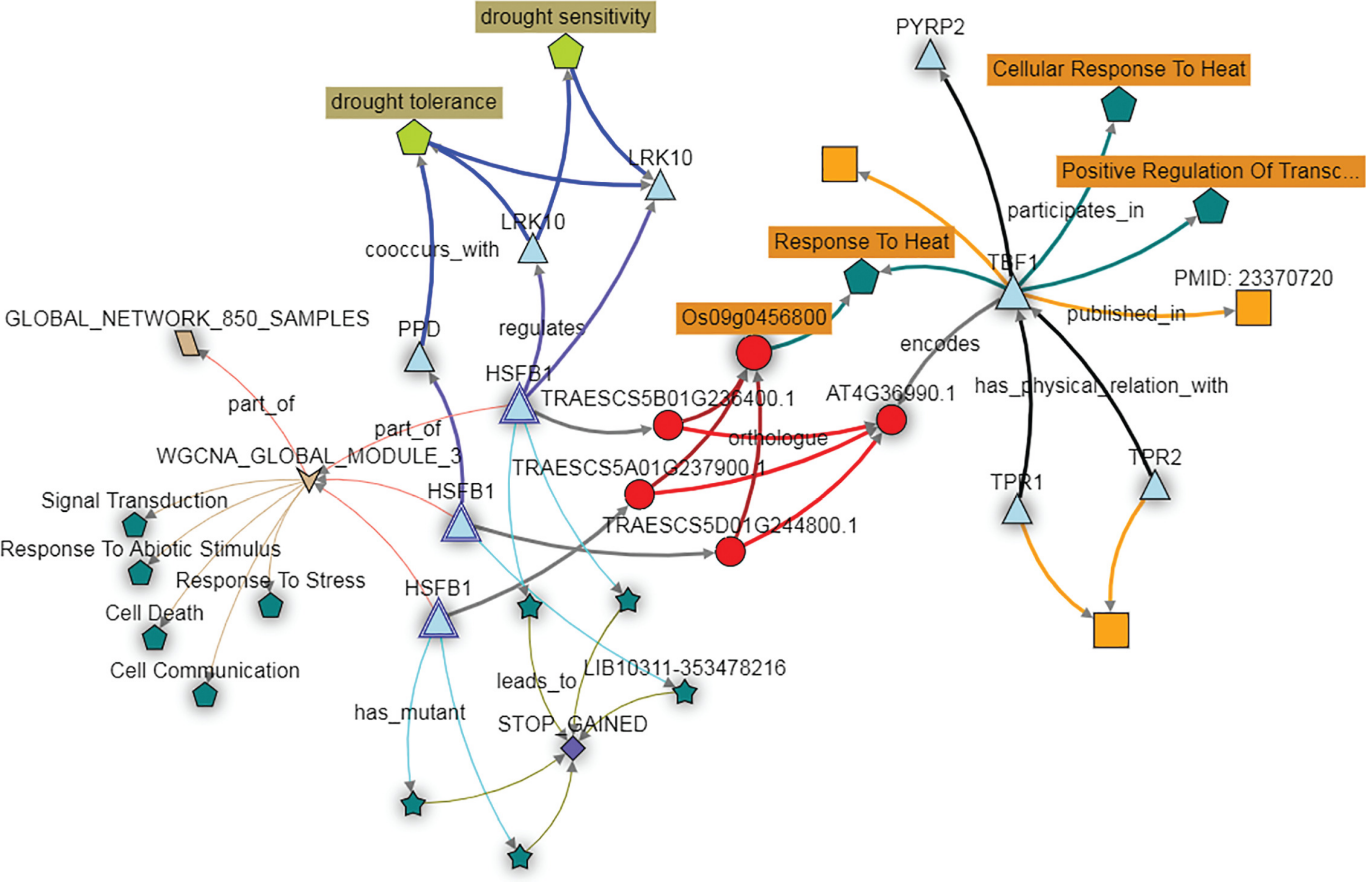
The KnetMiner network illustrates the putative role of the wheat *TBF1* orthologs in responding to abiotic stress. The wheat orthologs of the *Arabidopsis* gene *TBF1*, here depicted as three copies of the gene *HSFB1* (light blue triangles) fall in expression module three (brown arrow; WGCNA module 3). The genes in this module are enriched for GO terms such as ‘Response to Stress’ and ‘Response to Abiotic Stimulus’ (dark green pentagons). The *HFSB1* homoeologs are predicted to regulate other genes (blue triangles) in the GENIE3 network (purple connecting arrows) which are associated with the drought tolerance trait ontology terms (light green pentagon). PTC mutations are available for all three *HFSB1* homoeologs (dark green stars connecting with STOP GAINED SNP effect) in the Cadenza population.

Further investigation of these homoeologs can be performed using the KnetMiner knowledge network. For wheat *TBF1* orthologs, this includes homology, co-expression data, and associated TILLING mutants, combined with other wheat-specific information such as GENIE3 networks, wheat related publications, gene-phenotype relations extracted from the literature, GWAS data and *Arabidopsis* protein-protein interactions. Here the wheat genes, referred to as *HSFB1,* are orthologous to the *Arabidopsis* gene *TBF1* as demonstrated earlier, and the three wheat homoeologs fall into a module associated with responses to abiotic stresses (Figure 6). In addition, the *HSFB1* B and D homoeologs are predicted in the GENIE3 network to target the *LRK10* and *PPD* genes, which have known links to drought tolerance and sensitivity (Figure 6). The KnetMiner database also recapitulates the relationship between the wheat *HSFB1* homoeologs and their rice and *Arabidopsis* orthologs, which regulate heat stress responses (Figure 6). Considered as a whole, these data support the hypothesis that the *HSFB1* wheat genes are involved in the response to abiotic stress, perhaps specifically in drought response.

After evaluating *in silico* expression levels, we can then characterise the phenotype of wheat *TBF1* mutants using the exome-sequenced wheat TILLING mutant populations (Figure 2). We suggest to initially use the Kronos population, as it is based on a tetraploid line and thus contains only two copies of the gene (A and B homoeologs). This means that only two mutants need to be crossed to generate a full knockout. The hexaploid Cadenza TILLING population could also be used, but this would require an additional generation to combine mutant alleles across all three homoeologs (Figure 4).

All TILLING mutations identified against the more recent RefSeqv1.0 genome can be accessed directly from Ensembl Plants in the ‘Genetic Variation’ section. Available mutations in the gene of interest can be visualised as a table or positioned along the gene using the ‘Variant Image’ or ‘Variant Table’ option. We can thus rapidly identify mutations that are predicted to lead to a premature termination codon (PTC). However, if no appropriate PTC mutations are available, splice-site mutations predicted to lead to downstream frameshifts, or missense mutations in highly conserved amino acid residues with low SIFT (Sorting Intolerant from Tolerant; Ng and Henikoff, 2003) scores are good alternatives. SIFT scores predict the effect of a mutation on protein function and are based on the physical properties of the alternative amino acid as well as sequence homology.

For both the A and the B genome *TBF1* homoeologs in Kronos, no PTC mutations are available. However, we identified missense mutations in highly conserved residues with SIFT scores of 0 suggesting that these mutations are likely to have a deleterious effect on protein function (Figure 5D). In addition to SIFT, we also recommend using the PSSM viewer (https://www.ncbi.nlm.nih.gov/Class/Structure/pssm/pssm_viewer.cgi) to help predict the effect of specific missense mutations on conserved protein domains.

TILLING lines from both populations can be ordered via the GRU (https://www.seedstor.ac.uk/shopping-cart-tilling.php) in the UK or from the Dubcovsky lab (https://dubcovskylab.ucdavis.edu/wheat-tilling) in the USA. To maximise the chance of having selected functionally important mutants, we recommend choosing two independent mutant lines for each homoeolog and carrying out crosses between each mutant in the A and B genomes (four crosses shown in Figure 5D). Detailed guides on growing wheat plants, genotyping TILLING mutants, and crossing mutants can be found on www.wheat-training.com.

Seedlings are genotyped to confirm that the correct mutation is present and to select for homozygous individuals for crossing. To do this, we design genome-specific primers to use in a KASP assay as outlined above and on www.wheat-training.com. For most TILLING mutations genome-specific primers have been predesigned and are available in Ensembl Plants. If there are no suitable predesigned primers, online tools such as PolyMarker can be used (Ramirez-Gonzalez et al., 2015), or if needed, can be designed manually. After carrying out the initial cross, we grow the F_1_ individuals under speed breeding conditions, and self-pollinate to obtain the F_2_ seed. We then grow F_2_ individuals and select via genetic markers individuals homozygous for one or both mutant alleles, as well as homozygous wild type control individuals (Figure 4). We can then carry out our first phenotypic evaluation on the F_2_ plants using the homozygous wild type lines as controls without the need for backcrossing to Kronos. We can do this because the background mutations in the chosen lines will be segregating within both the mutant and the wild type lines, leading to an equivalent background mutation load between the sibling genotypes (Uauy et al., 2017). Backcrossing to Kronos can be started either with the single mutants while carrying out the initial cross and/or with the F_2_ double mutant at a later stage. Backcrossing to remove background mutations is especially important when studying quantitative traits, such as yield components (Simmonds et al., 2016), and when plants are intended for field phenotyping.

## Concluding remarks

In the last few years there has been a dramatic expansion in the resources available to carry out functional genomics in wheat, largely based upon improvements in the available reference sequences. Within a few years a step-change has been achieved from a highly fragmented assembly with incomplete gene models to a full pseudomolecule reference sequence alongside a detailed gene model annotation. This reference sequence allows the physical anchoring of genes in complete chromosomal order and provides improved gene models facilitating the design of transgenic constructs and primers. Most resources described in this review are integrated with the recent bread wheat reference genome sequence including the expVIP and eFP expression browsers, TILLING mutants, and Ensembl Plants sequence analyses and display tools. As a result, it is now easier to use these resources as they are unified by a common reference genome and gene models. Furthermore, a pan-genome of wheat is being produced which will provide high quality genome sequences for multiple varieties of wheat. These genomes will facilitate functional studies in a range of different genetic backgrounds and enhance the value of the populations containing natural variation captured from diverse wheat varieties.

## Future directions

Whilst many major advances have been made in the last five years to lay the groundwork for gene discovery and functional characterisation in polyploid wheat, looking to the future several key challenges remain.

- Polyploidy is a common challenge amongst crop species. In wheat, we frequently assume that due to functional redundancy it will be necessary to knock out all three homoeologs of a gene to assess its phenotypic impact. Yet the extent of homoeolog functional redundancy is still unclear (Borrill et al., 2019). Transcriptomics and proteomics approaches will help generate hypotheses as to the extent of homoeolog redundancy in wheat and allow researchers to specifically target the most phenotypically relevant homoeolog for genetic manipulation.
- Defining accessible (open) chromatin regions allows the identification of *cis*-regulatory sequences of potential functional significance. In animals and plants, genetic variants associated with quantitative traits are significantly enriched in these open chromatin sequences (Maurano et al., 2012; Rodgers-Melnick et al., 2016). In wheat, where over 98% of the genome is non-coding, it will be critical to identify open chromatin regions to more precisely define non-coding variation that may be of functional relevance. Work in tomato has elegantly shown how a wide range of phenotypic variation for quantitative traits can be engineered by genome editing of *cis-*regulatory regions of transcription factors (Rodríguez-Leal et al., 2017).
- To more readily test these hypotheses, increased transformation efficiency and reduced costs will also reshape the future of wheat research, perhaps one day becoming as accessible for wheat researchers as floral dip transformation is for *Arabidopsis*. It is becoming clear from research in wheat and other species that genetic background can have a strong influence on gene function. Therefore, it is essential to develop new protocols to transform multiple wheat varieties to account for these effects and to ensure that the potential of gene editing approaches is fulfilled.
- Genomic databases have been powerful in integrating data from multiple studies and international efforts are now bringing together phenotypic data alongside genotypic data (e.g. Blake et al., 2016 and Howe et al., 2020). Challenges remain to standardise phenotype collection protocols and ontologies, which will realise the full power of this information (e.g. Papoutsoglou et al., 2020). Expanding these databases to include environmental conditions will allow assessments of interactions between genotypes, phenotypes and the environment.

High quality genome sequences facilitate moving beyond gene-based analysis, revealing the effects of non-genic regions on phenotype. Whilst working in crops with complex genomes will remain challenging, the advance of genomic techniques has enabled the wheat community to leverage lessons learnt in model species. The approaches taken in wheat provide a framework to understand biologically important traits in other species with large genomes.
